# Fish consumption and risk of rheumatoid arthritis: a dose-response meta-analysis

**DOI:** 10.1186/s13075-014-0446-8

**Published:** 2014-09-30

**Authors:** Daniela Di Giuseppe, Alessio Crippa, Nicola Orsini, Alicja Wolk

**Affiliations:** Division of Nutritional Epidemiology, Institute of Environmental Medicine, Karolinska Institutet, Nobels vag 13, Stockholm, 171 77 Sweden

## Abstract

**Introduction:**

The association between fish consumption and rheumatoid arthritis (RA) is unclear. The aim of this paper was to summarize the available evidence on the association between fish consumption and risk of RA using a dose-response meta-analysis.

**Methods:**

Relevant studies were identified by a search of MEDLINE and EMBASE through December 2013, with no restrictions. A random-effects dose-response meta-analysis was conducted to combine study specific relative risks. Potential non-linear relation was investigated using restricted cubic splines. A stratified analysis was conducted by study design.

**Results:**

Seven studies (four case-controls and three prospective cohorts) involving a total of 174 701 participants and 3346 cases were included in the meta-analysis. For each one serving per week increment in fish consumption, the relative risk (RR) of RA was 0.96 (95% confidence interval (CI) 0.91 to 1.01). Results did not change when stratifying by study design. No heterogeneity or publication bias was observed. When fish consumption was modeled using restricted cubic splines, the risk of RA was 20 to 24% lower for 1 up to 3 servings per week of fish (RR =0.76, 95% CI: 0.57 to 1.02) as compared to never consumption.

**Conclusions:**

Results from this dose-response meta-analysis showed a non-statistically significant inverse association between fish consumption and RA.

## Introduction

Rheumatoid arthritis (RA) is an inflammatory autoimmune disease that affects the joints, leading to cartilage and bone destruction. The worldwide prevalence of this chronic disease ranges between 0.5% and 1.0% [1]. Smoking [2] and alcohol consumption [3,4] have been linked to the development of RA, but little is known about other modifiable risk factors. Among dietary factors, fish consumption is of particular interest due to its role in primary prevention of several chronic diseases, including cardiovascular disease [5,6]. Moreover, fish is rich in long-chain n-3 polyunsaturated fatty acids, which have been shown to be beneficial in primary [7] and secondary prevention [8] of RA.

The association of fish consumption with risk of developing RA is still unclear, because results from both case-control and cohort studies are mixed. One prospective cohort [7] and two case-control studies [9,10] have observed an inverse association between fish consumption and RA risk, but results were not statistically significant. On the other side, two prospective [11,12] and two case-control studies [13,14] did not show an association with total fish consumption.

The aim of the present study was to quantitatively summarize the published evidence from epidemiological studies on the association between fish consumption and RA using a dose-response meta-analysis.

## Methods

We conducted a literature search through December 2013 using PubMed and EMBASE databases. The term, rheumatoid arthritis, was used in combination with fish, or seafood. Reference lists from acquired articles were also examined. Studies were included in the meta-analysis if they met the following inclusion criteria: the exposure was fish or seafood consumption; the outcome was incident RA; relative risk (RR) or odds ratio (OR) estimates were reported with their 95% CI.

From each study we collected information on the first author’s last name, publication year, country, study period, number of cases and controls or cohort size, gender and age of study participants, covariables adjusted for, and RRs or ORs with 95% CI for each exposure category. If multiple RRs and ORs were presented, we extracted the estimates from the maximally adjusted model in order to reduce the risk of possible unmeasured confounding. Data extraction was performed independently by two of the authors (DDG and AC).

The quality of studies was assessed using the Newcastle-Ottawa quality assessment scale (NOQAS) for cohort and case-control studies, with which each study was judged based on the selection of the study groups, the comparability of the groups, and the ascertainment of exposure and outcome [15]. The score ranged between 0 (as poor) and 9 (as excellent). The present work follows the recommendations of the preferred reporting items for systematic reviews and meta-analyses (PRISMA) Statement [16]. This study did not need ethical approval or consent from patients.

### Statistical analyses

A two-stage dose-response random-effects meta-analysis was conducted to combine risk estimates [17-19]. The dose-response relationship curves were estimated by taking into account the covariance among risk estimates for different exposure categories [18]. The midpoint between the upper and lower boundary of each category was assigned to the corresponding risk estimate. For open-ended lowest categories, the lower bound was considered as zero, while the open-ended highest categories were assumed to be of the same amplitude as the preceding categories. For the study of Di Giuseppe *et al*. [7], the mean fish consumption within each exposure level was obtained from the primary data. Results from a study that reported only the linear association for grams per day of fish consumption were rescaled to servings per week [12].

A potential non-linear relation between fish consumption and RA risk was investigated using restricted cubic splines with three knots at fixed percentiles (10%, 50%, and 90%) of the exposure distribution. Departure from linearity was assessed by testing the null hypothesis that the coefficient of the second spline was equal to zero [20]. The study by Pedersen *et al*. was excluded from the flexible dose-response analysis because it reported information only on the linear trend [12].

In all meta-regression models, statistical heterogeneity between studies was evaluated with the Cochran *Q*-test and the *I*^2^ statistic [21] that assesses the proportion of total variation due to between-study variation. Publication bias was investigated by the Egger regression asymmetry test [22]. Sensitivity analyses were conducted by stratifying for study design and by excluding one study at the time to evaluate if results were particularly influenced by single studies. Statistical analyses were performed with R software, version 3.0.2, using the packages metafor and dosresmeta [23].

## Results

**Figure 1:**
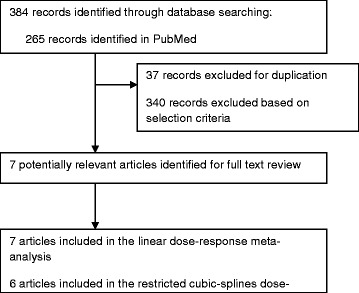
**Flow chart of selection of studies for inclusion in the dose-response meta-analysis.**

Of the 384 studies identified through PubMed and EMBASE, only seven examined the association between fish consumption and risk of RA and were included in this dose-response meta-analysis (Figure 1). Characteristics of the included studies are showed in Table 1. Three studies were prospective cohorts [7,11,12] and included 170,986 participants, of which 820 developed RA during the follow-up time (2,212,395 person-years), while four studies were of case-control design [9,10,13,14], of which two had hospital-based [9,13] and two had population-based controls [10,14], including a total of 2,526 cases and 3,715 controls. Three of the studies analyzed RA risk only among women [7,11,14], while the remaining analyzed both men and women [9,10,12,13]. Three studies adjusted only for age and gender [9,12,13], while other took into account other possible confounding factors, such as smoking [7,10,11] and total energy intake [7,11,14]. Only one study adjusted for other dietary factors, such as red meat and dairy product consumption [7]. Using the NOQAS quality assessment, the seven studies were assessed to have moderate quality.

**Table 1:** **Characteristics of studies on rheumatoid arthritis and fish consumption**

**Author, year**	**Study population, country, follow-up period/study period**	**Cases/cohort size or controls**	**Gender, age (years)**	**Relative risk/odds ratio (95% CI)**	**Controlled variables**	**NOQAS** ^**2**^
**Cohort**						
Pedersen *et al*., 2005 [12]	Diet, Cancer and Health cohort, Denmark, 1993 to 2001	69/56 691	Men and women, 50 to 64	Per 30 g/day: 0.91 (0.68, 1.23)	Age and gender	6
Benito-Garcia, 2007 [11]	Nurses’ Health Study, USA, 1980 to 2002	546/82 063	Women, 30 to 55	Q1 (median 0.07 s/d^1^): 1.00 (ref)	Age, total energy intake, body mass index, smoking, total lifetime breastfeeding	7
				Q2 (0.13 s/d): 0.94 (0.73, 1.23)		
				Q3 (0.17 s/d): 1.09 (0.81, 1.47)		
				Q4 (0.25 s/d): 1.06 (0.80, 1.40)		
				Q5 (0.44 s/d): 0.96 (0.72, 1.26)		
Di Giuseppe *et al*., 2013 [7]	Swedish Mammography Cohort, 2003 to 2010	205/32 232	Women, 54 to 89	<1 s/w^1^ nel 1987, <1 s/w nel 1997:	Age, cigarette smoking, alcohol intake, use of aspirin, red meat consumption, dairy food consumption, energy intake	8
				1.00 (ref)		
				≥1 s/w nel 1987, <1 s/w nel 1997:		
				0.78 (0.50, 1.22)		
				<1 s/w nel 1987, ≥1 s/w nel 1997:		
				1.01 (0.66, 1.56)		
				≥1 s/w nel 1987, ≥1 s/w nel 1997:		
				0.71 (0.48, 1.04)		
**Case-control**						
Linos *et al*., 1991 [9]	Hospital-based controls, Greece	168/137	Men and women, 24 to 89	1-2 s/m^1^: 1.00 (ref)	Age and gender	5
				4-10 s/m: 0.64 (0.38, 1.08)		
				12+ s/m: 0.37 (0.13, 1.05)		
Shapiro *et al*., 1996 [14]	Population-based controls, USA, 1986 to 1991	324/1245	Women, 18 to 64	<1 s/w: 1.00 (ref)	Reference age, reference year, education, race, total caloric intake	8
				1- <2 s/w: 0.87		
				(0.62, 1.21)		
				≥2 s/w: 0.92 (0.67, 1.25)		
Linos *et al*., 1999 [13]	Hospital-based controls, Greece	145/188	Men and women, 18 to 84	Q1 (median 3 s/w): 1.00 (ref)	Age and gender	5
				Q2 (4 s/w): 1.21 (0.64, 2.29)		
				Q3 (6 s/w): 0.90 (0.47, -1.71)		
				Q4 (10 s/w): 0.95 (0.46, 1.96)		
Rosell *et al*., 2009 [10]	Population-based controls (EIRA), Sweden, 1996 to 2005	1889/2145	Men and women, 18 to 70	Never/seldom: 1.00 (ref)	Age, residential area, smoking, gender	6
				1-3 s/m: 0.8 (0.7, 1.0)		
				1-7 s/w: 0.8 (0.6, 1.0)		

Information on fish consumption was collected using food frequency questionnaires in all studies. ^1^s/d = servings per day, s/w = servings per week, s/m = servings per month. NOQAS, Newcastle-Ottawa Quality Assessment Scale (score from 0 as poor to 9 as excellent).

**Figure 2:**
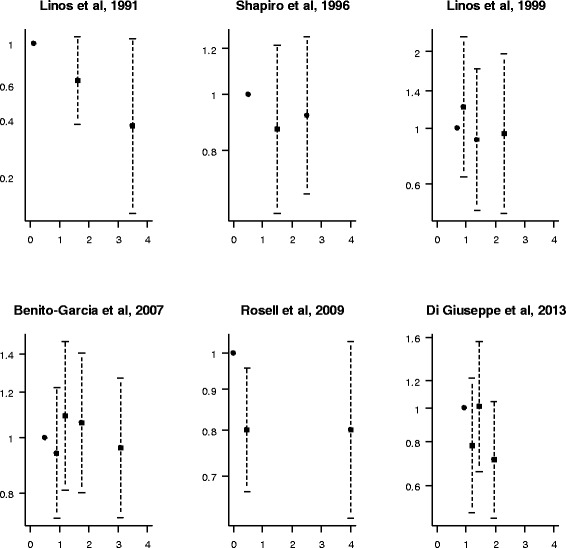
**Study-specific relative risk estimates for rheumatoid arthritis with increasing levels of fish consumption (servings per week).** Each panel refers to a study. Black squares indicate the relative risk estimates and whiskers their 95% CI. The vertical axis is on a log scale.

Three studies showed a borderline statistically significant inverse association between total fish consumption and risk of RA [7,9,10], while the others reported no association (Figure 2). Only two studies analyzed type-specific fish consumption [12,14]. A statistically significant inverse association was found between fatty fish and risk of RA in the study of Pedersen *et al*. [12], which, however, observed an increased risk for medium-fat fish consumption [12]. Broiled or baked fish was inversely associated with RA in the study of Shapiro *et al*. [14].

**Figure 3:**
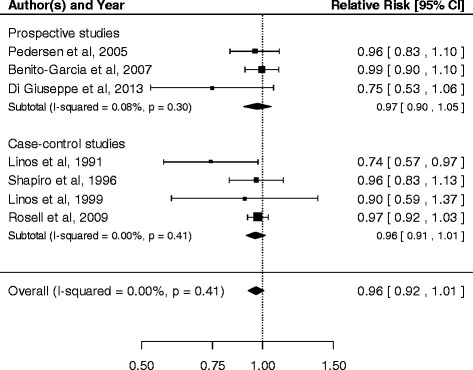
**Relative risk of rheumatoid arthritis for every one serving per week increase in fish consumption.**

We first assumed a linear-response model for the association between fish consumption and risk of RA. The risk of RA decreased by 4% for every increase of one fish serving per week, although this change was not statistically significant (RR =0.96, 95% CI 0.91, 1.01) (Figure 3). There was no evidence of heterogeneity across the studies (*I*^2^ = 0.0%, *P*-value =0.41) or of publication bias (*P*-value =0.27). Results stratified by study design were similar: the pooled RR was 0.96 (95% CI 0.91, 1.01) and 0.97 (95% CI 0.90, 1.05) for case-control and for cohort studies respectively. We also stratified by number of covariates considered: the pooled RR of the three studies that adjusted only for age and gender was 0.88 (95% CI 0.74, 1.05), while it was 0.97 (95% CI 0.93, 1.02) for the four studies that considered also covariates other than age and gender. A sensitivity analysis excluding one study at the time showed that the linear trend ranged between 0.95 (95% CI 0.91, 1.00) and 0.97 (95% CI 0.93, 1.01).

**Figure 4:**
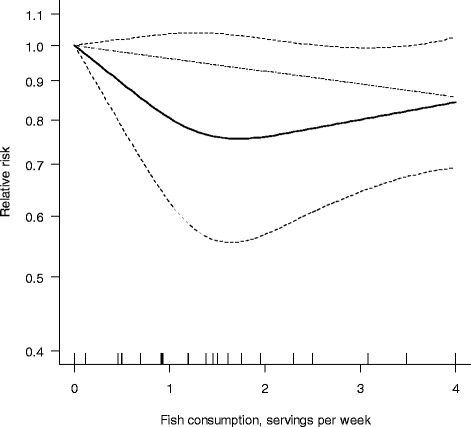
**Pooled dose-response association between fish consumption and rheumatoid arthritis risk (solid line).** Fish consumption was modeled with restricted cubic splines in a multivariate random-effects dose-response model. Dashed lines represent the 95% CI for the spline model. The dotted line represents the linear trend. Tick marks below the curve represent the positions of the study-specific relative risks. The value of 0 servings per week served as referent. The relative risks are plotted on the log scale.

We next allowed departure from linearity by fitting a spline model. We found no statistically significant departure from a linear association between fish consumption and RA risk (P_non-linearity_ =0.15) (Figure 4). However, the plot of the relative risk based on the flexible approach showed a decrease in risk for up to two servings per week, followed by a slight increase in risk for higher consumption. Compared with no fish consumption, one, two and three servings per week were associated with 20% (RR =0.80, 95% CI 0.62, 1.04), 24% (RR =0.76, 95% CI 0.57, 1.02) and 20% (RR =0.80, 95% CI 0.65, 0.99) lower RA risk, respectively.

## Discussion

The present study is the first dose-response meta-analysis on the association between fish consumption and risk of developing RA. Results from this meta-analysis showed a weak inverse association between total fish consumption and risk of RA. The flexible non-linear approach for modeling fish consumption showed a decrease in RA risk for one to three servings per week of fish consumption, followed by a slight increase in risk for higher consumption.

The inverse association between fish consumption and risk of developing RA observed in some of the studies has been mainly attributed to its content of long-chain n-3 polyunsaturated fatty acids (PUFAs) [7,10,14]. Indeed, when long-chain n-3 PUFAs are included in the analysis model the inverse association between fish and RA disappears [7]. A possible reason behind this inverse association is the anti-inflammatory properties of these fatty acids. In fact, the n-3 PUFA eicosapentaenoic acid (EPA) and docosahexaenoic acid (DHA) are metabolized to competitive inhibitors of n-6 PUFAs (prostaglandins and leukotrienes) and suppress the production of the inflammatory cytokines [24]. The lack of a statistically significant inverse association observed in this meta-analysis could be explained by the balance of the protective effect of long-chain n-3 PUFAs with the presence of contaminants such as polychlorinated biphenyls (PCBs), which have been found to be positively associated with RA [25]. Moreover, the presence of PCBs could explain why in the study of Shapiro *et al*., the inverse association was statistically significant only for broiled or baked fish and not for total fish consumption, as the presence of PCBs is lower in cooked fish [26].

The main strength of this meta-analysis is its dose-response design, that provides better quantification of the associations between specified amounts of fish and risk of RA. A dose-response meta-analysis should be the preferred option when performing a systematic review, rather than running a meta-analysis based only on the comparison of the extreme categories of consumption (high versus low), which could vary considerably among studies.

Among the limitations, a meta-analysis is deeply influenced by the quality of the single studies included. Results could be over- or underestimated due to residual confounding. Of the seven studies included in this meta-analysis, three [9,12,13] adjusted only for age and gender, and therefore their results could be affected by residual confounding by factors such as smoking and total energy intake. This meta-analysis summarizes results from both prospective cohort and case-control studies. However, these two types of study design have many differences, including the different statistical estimate (hazard ratio versus odds ratio) and different biases. In fact, despite all studies using a food frequency questionnaire (FFQ) to assess fish consumption, participants in prospective cohort studies received the FFQ when they were disease-free, while in case-control studies, the cases received the FFQ after diagnosis, leading to a possible recall bias. Also, the RA diagnosis could be misclassified. Of the seven studies included in the meta-analysis, two prospective cohort studies identified cases by linkage with national health registers [7,12], while one prospective cohort used self-reported RA cases that were subsequently validated [11], a method that could have led to under-detection of cases who did not self-report RA. Due to the limited number of studies, it was not possible to stratify according to country to evaluate if the differences in fish consumption between countries could have influenced the results of the meta-analysis. Moreover, there were also differences in the representativeness of the general population in the single studies: studies from Scandinavia [7,10,12] represented their respective general populations well, while the level of representativeness of the other studies is less clear.

## Conclusion

In conclusion, the summary estimates from this dose-response meta-analysis show a non-statistically significant inverse association between fish consumption and RA.

## Abbreviations

FFQ: food frequency questionnaire
NOQAS: Newcastle-Ottawa quality assessment scale
OR: odds ratio
PCB: polychlorinated biphenyl
PUFA: polyunsaturated fatty acid
RA: rheumatoid arthritis
RR: relative risk
